# Quality of acute ischemic stroke care at a tertiary Hospital in Ghana

**DOI:** 10.1186/s12883-021-02542-9

**Published:** 2022-01-17

**Authors:** Frank Kumi, Amos A. Bugri, Stephen Adjei, Elvis Duorinaa, Matthew Aidoo

**Affiliations:** 1 Pharmacy Unit, King’s Medical Center, Tamale, Ghana; 2 Pharmacy Directorate, Tamale Teaching Hospital, Tamale, Ghana; 3 Department of Pharmacology, University for Development Studies, Tamale, Ghana

**Keywords:** Quality indicators, Acute ischemic stroke, Performance measurement, Outcome and processes measures

## Abstract

**Background:**

Information on the quality of acute ischemic stroke care provided in lower-to-middle income countries is limited.

**Objective:**

This study was undertaken to examine the quality of acute ischemic stroke care provided at Tamale Teaching Hospital in Ghana.

**Methods:**

The medical records of patients admitted into the medical ward of the hospital between January to October 2021 were reviewed retrospectively. Extent of compliance to 15 stroke performance indicators were determined.

**Results:**

Under the study period, 105 patients were admitted at the hospital with acute ischemic stroke. The mean (±SD) age was 65 ± 12 years; 38.1% were males; 65.7% had National Health Insurance Scheme coverage. Glasgow Coma Scale was the only functional stroke rating scale used by physicians to rate stroke severity. About a quarter of the patients had CT scan performed within 24 h of admission. Less than a quarter of the patients had a last known well time documented. Rate of thrombolytic administration was 0%. Less than a quarter of the patients were prescribed venous thromboembolism prophylaxis on the day of admission or day after. Only 13.8% of patients had documented reasons for not being prescribed venous thromboembolism prophylaxis. Antiplatelet therapy was prescribed to 33.3% of the patients by the end of day 2 of admission. Anticoagulation was prescribed to all patients who had comorbid condition of atrial fibrillation as part of the discharge medications. More than half of the patients were discharged to go home with statin medications. Documented stroke education was provided to 31.4% caretakers or patients. Slightly less than half of the patients were assessed for or received rehabilitation. Less than a quarter had documented dysphagia screening within 24 h of admission. None of the patient had their stroke severity rated with National Institutes of Health Stroke Scale on arrival. No patient obtained carotid imaging assessment by end of day 2.

**Conclusion:**

There were several gaps in the quality of acute ischemic stroke care provided to patients at the Tamale Teaching Hospital. With the exception of discharging patients on statin medications, there was poor adherence to all other stroke performance indicators.

## Background

Across the world, stroke has been recognized as a leading cause of mortality and morbidity. Stroke is among the top-ranked causes of disability-adjusted life-years (DALY) in the middle aged and the elderlies [1]. The risk of cerebrovascular disease is higher in low-to-middle income countries owing to barriers to stroke care [2]. In Africa, the burden of stroke appears to be increasing as modeled-based estimates have shown significant mean increases in age-standardized stroke incidence [3]. In Ghana, the trends in stroke admissions and mortality has been gradually increasing over the last 30 years with a 28-day mortality rate as high as 41% in the central part of Ghana, a worrying phenomenon which require aggressive risk modification and improvement in the quality of acute stroke care [4]. Furthermore, the burden of stroke is reported to be increasing with a one-month in-hospital case fatality as high as 41–43% [5].

Development and implementation of stroke performance measures has been widely advocated as part of measures to bridge the gap between evidence-based clinical practice guidelines recommendations and routine clinical practice. To reduce the global burden of cerebrovascular diseases, stroke key performance indicators have been developed for adaptation depending on level of health service capacity [2, 6]. Large-scale improvement in stroke care can be achieved through the adoption and implementation of performance measures [7]. Researchers in both advanced and developing countries have attempted to measure the quality of care provided to stroke patients as part of quality improvement initiatives towards stroke care [8–10].

Routine activities of clinical practice which aligns with guidelines-directed recommendations can have an overall favorable effect on patient outcomes through continuous quality improvement efforts. Adherence to key stroke performance indicators which are based on stroke clinical practice guidelines (swallowing assessment, stroke unit admission, antiplatelet for ischemic stroke admission etc.) have been shown to be associated with a reduction in the risk of death or disability from stroke [11, 12].

The data on stroke quality care is limited. Although a previous study has examined the quality of stroke services provided in major referral hospitals across the country [5], this study was limited by its reliance on responses from respondents through a cross-sectional survey with the potential of gathering results which could not be reflective of the real situation on the ground. The Tamale Teaching Hospital (TTH) is the major referral facility for the northern part of Ghana. In a study of the mortality patterns at the Accident and Emergency Department of the hospital, cerebrovascular accident (CVA) ranked third in the causes of death at the department [13]. This signifies an opportunity to monitor and improve the quality of care provided to patients who suffer from CVA at the facility.

This study was conducted to examine the quality of ischemic stroke care based on the World Stroke Organization Global Stroke Services Guidelines and Action Plan [2] as well as stroke performance measures of the American Heart Association/American Stroke Association (AHA/ASA) Clinical Performance Measures [6] provided at the Tamale Teaching Hospital. Overall, compliance with 15 performance indicators spanning the dimensions of stroke management (assessment and diagnosis, acute management and secondary prevention) were assessed. This would provide information to help fill some of the knowledge gap in stroke quality care in addition to serving as the foundation towards organized stroke care aimed at improving the quality of clinical care provided to stroke patients at the TTH.

## Methods

### Study site

Selected site for the study was the medical wards of the TTH. Provision of care were done by a diverse group of health professionals [specialist medical doctors (2): endocrinologist and gastroenterologist; medical doctors (6); nurses (100); pharmacists (5) and physiotherapists (4)]. Located in the northern part of Ghana, TTH is one of 5 public teaching hospitals in the country. With a current bed capacity of 800 beds, the hospital provides diverse specialist services to people in the Savanna regions including the Upper West and East regions, Northern Region, some parts of the Brong–Ahafo region and the northern parts of the Volta region of Ghana [14]. In summary, the hospital provides services to over 100,000 patients each year as a major referral center in the northern part of the country [14].

### Study design

We conducted a cross-sectional retrospective review of the medical records of patients with stroke.

### Source population

The population source of this study were patients on admission at the medical wards of TTH between the periods of 1st January, 2021 to 31st October, 2021.

### Study population

All patients with documented diagnosis of stroke admitted into the medical wards of TTH from 1st January to 31st October, 2021 constituted the study population.

### Selection criteria

The following inclusion criteria were considered: i) patients with medical records documenting a diagnosis of ischemic stroke, complete patient characteristics, and treatment outcomes.

Exclusion criteria considered were: i) patients with diagnosis of hemorrhagic stroke ii) patients with ischemic stroke with incomplete patient characteristics, or without treatment outcomes and iii) patients discharged against medical advice.

### Sample size and sampling technique

All patients who met the eligibility criteria were included in our study due to the small sample size of our study participants.

### Study variables

**Fig. 1:**
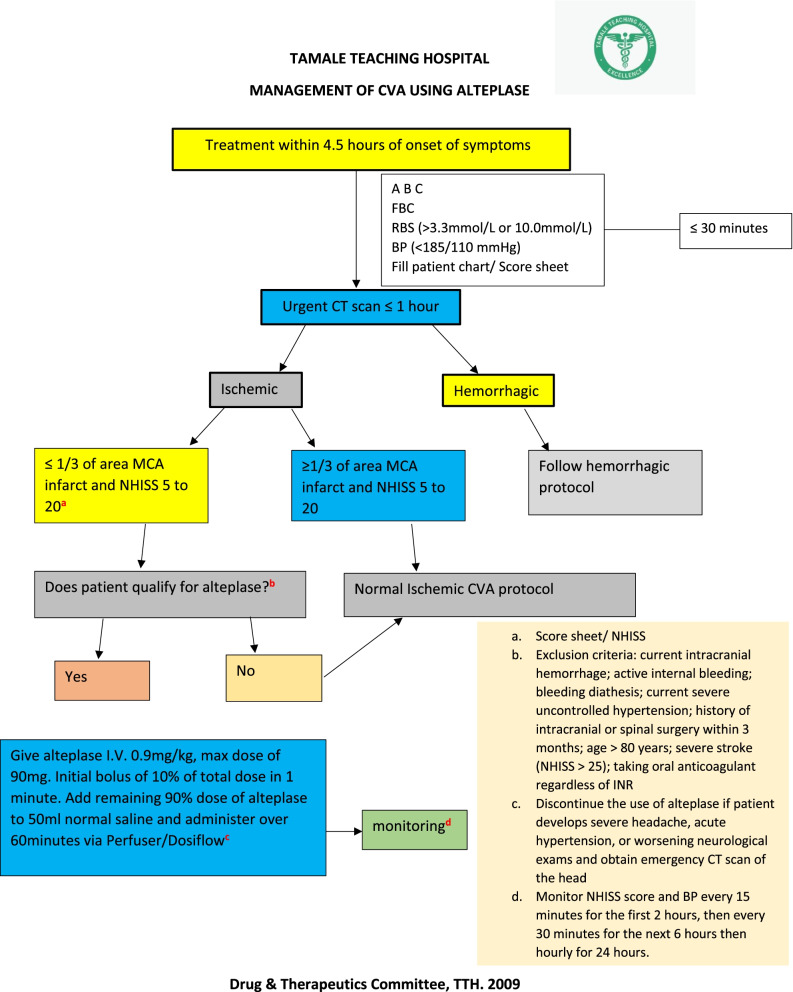
The Tamale Teaching Hospital protocol for the management of ischemic stroke

Criteria for diagnosis of ischemic stroke: An episode of neurological dysfunction caused by focal cerebral, spinal or retinal infarction based on i) pathological, imaging [e.g. computated tomographic (CT) scan, magnetic resonance imaging (MRI) etc.], or other objective evidence [15] or ii) an episode of neurological dysfunction caused by focal cerebral, spinal or retinal infarction based on clinical symptoms (e.g. hemiparesis, monoparesis, quadriparesis, facial drop, slurred speech, retinopathy etc.) persisting ≥24 h or until death and other etiologies excluded [16]. TTH has a protocol for the diagnosis and management of stroke at the facility (Fig. 1).

### Data collection

Data extracted from patients’ medical record included demographic information, stroke risk factors, stroke severity and health insurance plan. In addition to these, the following stroke performance indicators as adapted from the World Stroke Organization Global Stroke Services Guidelines and Action Plan [2] as well as stroke performance measures of the American Heart Association/American Stroke Association (AHA/ASA) Clinical Performance Measures [6] were collected by trained research assistants with the aid of standardized data collection tool: computed tomography (CT) scan performed within 24 h of admission; time (hours) last known-well documented; last known-well ≤4.5 h; thrombolytic therapy provided if patient presented within 4.5 h of last known well; antiplatelet prophylaxis on day of admission or day after admission; documented reasons for not prescribing antiplatelet prophylaxis; antithrombotic therapy prescribed by end of day 2; discharged on antithrombotic therapy; atrial fibrillation or flutter patients discharged on anticoagulation; discharged on a statin medication; documented stroke education provided to caretakers or patients; assessed for or received rehabilitation/physiotherapy; documented stroke education provided to caretakers or patients; National Institutes of Health Stroke Scale (NIHSS) on arrival; documented dysphagia screening within 24 h of admission; carotid imaging assessment by end of day 2.

### Data analysis

Statistical Package for the Social Sciences (SPSS) Version 22.0 was used to analyzed the data. All data, including demographics, baseline characteristics and performance measures were analyzed with descriptive statistics of frequency such as mean (± standard deviation and data summarize into percentages were presented into tables.

### Ethical consideration

Approval to collect data was sought from the hospital’s Department of Research.

## Results

Between January 2021 to October 2021, 105 patients were admitted at the hospital with acute ischemic stroke (AIS) as the confirmed diagnosis duly documented in the patients’ medical records. The social demographics of the patients showed a mean (±SD) age of 65 ± 12 years. More than half of these patients were females (approximately 62%) (Table 1). It is observed that 65.7% of the patients had National Health Insurance Scheme (NHIS) coverage, whilst 34.3% exclusively paid out of pocket for their medical services. A review of the patients’ clinical characteristics for vascular risk factors for stroke indicated that hypertension was the highest (88.6%), followed by diabetes mellitus (38.1%), previous stroke (12.4%), dyslipidemia (7.6%), previous TIA (4.8%), atrial fibrillation/flutter (2.9%), and smoking (1.9%) being the least as shown in Table 1. Glasgow Coma Scale (GCS) was the only functional stroke rating scale used by physicians to rate stroke severity. The GCS was grouped into three levels as mild, moderate and severe [17]. Majority of the patients admitted were in the mild stroke category (56.2%) per GCS scoring.

**Table 1:** Baseline characteristics of participants (*N* = 105)

Characteristic	Frequency	Percent (%)
Social factors		
Age, years (mean ± SD)	65 ± 12	
Male gender	40	38.1
Female gender	65	61.9
National Health Insurance Scheme coverage	69	65.7
Non- National Health Insurance Scheme coverage	36	34.3
Vascular risk factors		
Hypertension	93	88.6
Diabetes mellitus	40	38.1
Previous stroke	13	12.4
Dyslipidemia	8	7.6
Previous TIA	5	4.8
Atrial fibrillation or atrial flutter	3	2.9
Smoking	2	1.9
Stroke severity on admission		
GCS 13–15 (mild)	59	56.2
GCS 9–12 (moderate)	26	24.8
GCS 3–8 (severe)	20	19.0

From our review, 25/105 (23.8%) of the admitted patients with stroke had CT scan performed within 24 h of admission (Table 2). Less than a quarter of the patients 21/105 (20.0%) had a last known well time documented. Out of the patients with last known well time documented, 3/21 (14.3%) were last known well within ≤4.5 h (i.e. symptom of ischemic stroke occurred ≤4.5 h). However, these patients were not administered any thrombolytic therapy (Table 2). Only about 17% (18/105) of the patients were prescribed venous thromboembolism (VTE) prophylaxis on the day of admission or day after. Out of the 87 patients not prescribed VTE prophylaxis, only 13.8% (12/87) had documented reasons for not being prescribed VTE prophylaxis. Antiplatelet therapy was prescribed to 33.3% (35/105) of the patients by the end of day 2 of admission. Additionally, from the patients discharged, about 32.0% (30/94) were prescribed antiplatelets as part of their discharged medications (Table 2). All patients with atrial fibrillation/flutter as a comorbid condition were prescribed anticoagulants on discharge. More than half of the patients 56/94 (53.3%) were discharged on statin medications. Documented stroke education were provided to less than one-third (31.4%) of caretakers or patients. Slightly less than half of the patients (about 43%) were assessed for or received rehabilitation/physiotherapy. Less than one-fourth of the patients (19.0%) had documented dysphagia screening within 24 h of admission. None of the patients obtained carotid imaging assessment by end of second day of admission. Again, none of the patients had their stroke severity rated with National Institutes of Health Stroke Scale (NIHSS) on arrival. Majority of the patients (89.5%) were discharged, however 11/105 (10.5%) died on admission (Table 3).

**Table 2:** Quality of ischemic stroke care parameters

Indicators	Frequency	Percent (%)
CT scan performed within 24 h of admission, N = 105	25	23.8
Time (hours) last known-well documented, N = 105	21	20.0
Last known well ≤4.5 h, *N* = 21	3	14.3
Thrombolytic therapy provided if patient presented within 4.5 h of last known well, *N* = 3	0	0
VTE prophylaxis on day of admission or day after, N = 105	18	17.1
Documented reasons for not prescribing VTE prophylaxis, *N* = 87	12	13.8
Antiplatelet therapy prescribed by end of day 2, N = 105	35	33.3
Discharged on antiplatelet therapy, *N* = 94	30	31.9
Atrial fibrillation or flutter patients discharged on anticoagulation, N = 3	3	100.0
Discharged on a statin medication, N = 105	56	53.3
Documented stroke education provided to caretakers or patients, N = 105	33	31.4
Assessed for or received rehabilitation/physiotherapy, N = 105	45	42.9
Documented dysphagia screening within 24 h of admission, N = 105	20	19.0
Carotid imaging assessment by end of day 2, N = 105	0	0.0
National Institutes of Health Stroke Scale (NIHSS) on arrival, N = 105	0	0.0

**Table 3:** Outcome measures

Outcome	Number of patients	Percent (%)
Discharged	94	89.5
Died before discharge	11	10.5

## Discussion

This study sheds light on the quality of care provided to ischemic stroke patients at the TTH, a tertiary hospital located in the northern part of Ghana. Generally, findings from this paper indicated gaps in quality of care across the various dimensions of stroke management: assessment, diagnosis, acute management, secondary prevention and rehabilitation.

Non-contrast-enhanced computed tomography (NECT) is recommended to be done within 25 min of patient’s arrival at the emergency department (ED) in order to facilitate timely administration of intravenous fibrinolytic therapy [16]. In this study, only about 25 (23.8%) of patients had their CT-scan results available within 24 h from the time of hospital admission. Out of these patients, only 3 had their results available within an hour of presentation to the hospital. We found out that throughout the study period, the hospital’s CT scan equipment was intermittently non-functional, hence patients often had to undergo CT imaging at private diagnostic centers outside the hospital. This obviously hampered timely evaluation and management of patients who presented with stroke. An opportunity therefore exists to improve the quality of care provided to stroke patients. Regular maintenance of the CT scan machine in addition to seamless integration of the neuroimaging center of the hospital into the care process for stroke patients can be explored by the facility management. Poor access to stroke diagnostic and assessment services across the country, particularly in the northern part has earlier been reported [5] and indeed this finding is consistent with results from another resource constraint country in West Africa [18].

Only less than a quarter of the patients whose medical records were reviewed had a clear last time known well documented in their medical record. Additionally, there were no standardized format of documenting this information. Recommendations regarding specific ways of documenting the date and time last known well have been proffered to ensure uniformity in documenting the time of stroke symptoms [19]. This important historical information could be obtained from the patient, care giver or bystander in the event that the patient is suffering from speech impairment [16]. To facilitate the accuracy of this information and to further enhance the continuity of care and timely interventions provided to stroke patients, a standardized format of documenting this fact can be explored and clearly reported in an unambiguous way in patients’ medical record [20–22]. To improve documentation, a standardized approach in a form of a protocol could be developed and implemented as this has been demonstrated to improve accuracy and documentation of this critical information during history taking in stroke patients [20].

Of the 21 patients who had properly documented time of symptom onset to presentation at the hospital, only 3 patients presented within 4.5 h from the time last known well. The excessive delay of stroke patients, especially in lower income countries, in presenting to the hospital for timely treatment has been widely acknowledged as well as the underlying barriers to timely intervention have been similarly explored [16, 23, 24]. Central to these barriers are lack of awareness of signs and symptoms of stroke as well as unavailability of emergency medical services [25]. Health promotion together with mass public health campaigns aimed at improving community awareness about the signs and symptoms of stroke could explored. This particular strategy has been reported to improve information seeking behavior, emergency admission and enhance the rate of thrombolytics administration [26].

Out of the 21 patients who had a well-established date and time of last known well documented, 3 of them presented within 4.5 h of symptoms onset. Therefore, these patients were probably eligible for thrombolytic therapy using the time criteria for administration of thrombolysis, because evidence shows that thrombolysis is effective within 4.5 h and probably a little more of ischemic stroke symptoms onset at any age and stroke severity in the absence of contraindications [16]. However, none of these patients received thrombolytic therapy and there was no documented reason (e.g., contraindication) for not administering the drug. A possible reason for this observation could be attributed to financial constraints of the patients owing to the high cost of these drugs. It is instructive to point out that a thrombolytic drug (alteplase) was available in the hospital during the study period. Therefore, no single patient received an administration of this critical medicine which is lauded as central to the improvement in functional outcomes in ischemic stroke patients. In developing countries, thrombolysis rate reported have been relatively low compared to developed countries. Whereas a prospective study in Thailand recorded 3.8% of ischemic stroke patients receiving intravenous recombinant tissue plasminogen activator (rtPA) [8], comparatively higher values ranging from 7.9 to 24% have been recorded in advanced countries like Germany, Spain etc. [27]. Various systematic interventions aimed at improving the rate of thrombolytics uptake can be examined and implemented. It is however important to tackle fundamental issues as such delay in presentation to hospital as well as early recognition of signs and symptoms of the disease. Subsequently, factors related to in-hospital delays, absence of a fully functional designated stroke unit could be addressed through interventional programs as has already been explored in other settings [28, 29]. A national policy intervention to address the high cost of rtPA needs to also be developed since high cost of rtPA has been noted as one of the key barriers to provision of standard of care to ischemic stroke patients [24].

Stroke raises the risk of venous thromboembolism by three (3) fold [30]. The incidence of deep vein thrombosis (DVT) and pulmonary embolism (PE) after stroke has been estimated as ranging from 1 to 80% [31]. Both DVT and PE are responsible for almost 10% of mortality after stroke [6]. Interventions for prevention of DVT include intermittent pneumatic compression (IPC) [32] and subcutaneous administration of anticoagulants. There are recommended for immobilized stroke patients [16]. Although IPC has been suggested to be safer and also effective compared to administration of anticoagulants, IPC services are not available in the TTH, thus the hospital relies on anticoagulant therapy for DVT and PE prophylaxis in ischemic stroke patients. In this study about 17% of ischemic stroke patients were prescribed medications for prophylaxis against venous thromboembolism (VTE). This is in sharp contrast to a similar study which reported that almost all patients had DVT prophylaxis by end of hospital day-2 [8]. The reasons for the low compliance with respect to this indicator at TTH could be many. One key underlying factor could be the absence of a dedicated stroke care unit and physicians with specialized knowledge and skills in stroke care management**.** The presence of stroke care units is associated with increased adherence to guideline-directed treatment recommendations, including the early administration of DVT prophylaxis for stroke patients [33].

Intracranial atherosclerosis is a major cause of stroke globally, accounting for high rate of stroke recurrence [34]. The administration of antiplatelet drug e.g. an initial dose of 325 mg of aspirin within 24 to 48 h of ischemic stroke is recommended because available evidence indicate a statistically significant decrease in mortality and negative outcomes with the administration of oral antiplatelets within 48 h after stroke [16]. Only about a one-third of the patients were prescribed early antiplatelets and discharged on them, a relatively low rate compared to relatively high rates reported in other studies [8, 10, 35]. This wide gap between this guidelines-directed recommendation and clinical practice at the TTH presents an opportunity for ischemic stroke care improvement through continuous professional development programs aimed at physicians and clinical pharmacists involved in the patient care process. Interventions instituted by pharmacists can increase adherence to guidelines recommendations on the need to prescribe antiplatelets to patients who have indications for it, including ischemic stroke patients with no contraindication [36].

In this study, three patients presented with atrial fibrillation/flutter. Prescribing oral anticoagulation is recommended in the absence of any contraindication in ischemic stroke patients with atrial fibrillation/flutter [16, 34]. All the patients were discharged on oral anticoagulation, representing 100% compliance with this performance measure. Higher compliance has been reported by similar studies [8, 10, 35]. Nevertheless, care should be taken in interpreting this result owing to a smaller sample size.

Compliance to prescribing a statin to ischemic stroke patients irrespective of the cause of the stroke was high. More than half of the patients received a statin prior to discharge. Statin prescribing to ischemic stroke has been variable in the literature. Whilst low compliance was reported in a study in China [35], higher rate of statin prescribing was realized in similar studies in Brazil and Thailand [8, 10]. Easy access and affordability to statins could be accounting for the high compliance since some of the statins are part of the medication benefit package under the Ghana National Health Insurance Scheme.

The importance of life-style related counselling in secondary prevention of ischemic is well documented and as such healthcare providers need to advise patients or care givers on the potential benefits of restriction of daily salt intake and following Mediterranean-diet [34, 37]. Less than a one-third of the patients or caretakers were provided documented counselling on the importance of life-style management. In addition to dietary advise, people who survive stroke are encouraged to also increase their level of physical activity to an extent permitted by their circumstances and if possible, attain physical activity goals that apply to the normal the population [34, 38].

About 43% of the patients were assess for and/or received early mobilization. This finding is surprising, given that this institution has a functional physical therapy department. This notwithstanding, with a carefully coordinated quality improvement efforts, this performance indicator could be improved. Not only does early mobilization reduce some of the complications linked with stroke [16], it has also been found to impact favorably on functional outcomes after stroke [39].

Swallowing assessment prior to oral administration of food or medication is recommended as a simple water swallow test which show the presence of a wet voice portends a high risk of aspiration [16]. The findings from this study indicate that only 19% of the ischemic stroke patients (less than a quarter) had a documented dysphagia screening within 24 h of admission. Clinicians at the hospital need to improve on the performance of this simple and important test since dysphagia screening test has no cost implications to the patient. Nurses trained to screen for dysphagia in stroke patients have been found to reduce stroke-related complications such as pneumonia [40].

Carotid imaging after stroke is recommended to identify carotid artery disease which remains an important and treatable cause of ischemic stroke [34]. None of the patient in this study was taken through carotid imaging, a finding in contrast to the results reported by another study in China where 64% of the patients received carotid imaging [35]. This finding could be attributed to lack of knowledge and availability of physicians with specialized knowledge in stroke management. Doppler ultrasound cervical carotid imaging is a cost-effective and non-invasive imaging technique recommended among other techniques to be used to screen for stenosis [41]. The TTH management need to implement measures to promote access to common non-invasive imaging techniques and also train care providers.

None of the patient, under the period of review, was taken through the NIHSS assessment. Although this study did not explore the barriers to the uptake of this outcome measurement tool, the underlying reason could be due to limited knowledge. It was found that most of the time, the GCS was the preferred functional rating scale. Nevertheless, there is a need to highlight that the NHISS has been found to be more accurate than the GCS [42] and it is indeed the preferred neurological assessment tool since it provides benefits which among other things include quantification of the extent of deficit in addition to the facilitation of communication [16].

This study has provided insights into some of the gaps associated with the management of ischemic stroke at the TTH, a tertiary hospital strategically situated in the country to address some of the complex health needs of the people living in the northern part of the country. The non-availability of functional stroke unit and as well as a Neurologist in the TTH is a possible reflection of the gaps observed in our study in management of stroke patients. In spite of this, certain key limitations need to be taken into account in interpretation the findings from the study. Since this is a retrospective study, data abstraction depended on availability of information documented in patients’ medical record. Consequently, it is possible that some health care interventions were administered by health providers but were not documented. For example, doctors or nurses who administered swallowing screening tests or provided counselling to patients or their care takers but failed to document these interventions would lead to low performance measurement scoring. Furthermore, this study did not delve into the evaluation of any association between the various performance indicators and functional stroke outcome or mortality.

## Conclusion

There were several gaps in the quality of acute ischemic stroke care provided to patients at the TTH. With the exception of adherence to the need to discharge patients on statin medications, there was poor adherence to all other stroke performance measures across the various dimensions of ischemic stroke care. The management of the hospital could consider supporting clinicians like doctors, physiotherapists, pharmacists and nurses to pursue specialization programs in the field of neurology as well as establish a functional stroke unit as this can help improve stroke management in TTH.

## Data Availability

The datasets generated and/or analysed during the current study are not publicly available due to the necessity to ensure patient confidentiality policies and laws of the country but are available from the corresponding author on reasonable request.

## Abbreviations

Afib: Atrial fibrillation
AHA/ASA: American Heart Association/American Stroke Association
CVA: Cerebrovascular accident
CT: Computated tomography
DVT: Deep vein thrombosis
GCS: Glasgow Coma Scale
NHIS: National Health Insurance Scheme
NIHSS: National Institutes of Health Stroke Scale
NECT: Non-contrast-enhanced computed tomography
PE: Pulmonary embolism
rtPA: Recombinant tissue plasminogen activator
TTH: Tamale Teaching Hospital
VTE: Venous thromboembolism
